# Ultra-processed food consumption in adults across Europe

**DOI:** 10.1007/s00394-021-02733-7

**Published:** 2021-12-03

**Authors:** Elly Mertens, Chiara Colizzi, José L. Peñalvo

**Affiliations:** Unit of Non-Communicable Diseases, Department of Public Health, Institute of Tropical Medicine, Antwerp, Belgium

**Keywords:** Ultra-processed foods, Food consumption survey, Europe, NOVA classification, Diet quality

## Abstract

**Purpose:**

The purpose of this study is to describe ultra-processed food and drinks (UPFDs) consumption, and associations with intake of total sugar and dietary fibre, and high BMI in adults across Europe.

**Methods:**

Using food consumption data collected by food records or 24-h dietary recalls available from the European Food Safety Authority (EFSA) Comprehensive European Food Consumption Database, the foods consumed were classified by the level of processing using the NOVA classification. Diet quality was assessed by data linkage to the Dutch food composition tables (NEVO) and years lived with disability for high BMI from the Global Burden of Disease Study 2019. Bivariate groupings were carried out to explore associations of UPFDs consumption with population intake of sugar and dietary fibre, and BMI burden, visualised by scatterplots.

**Results:**

The energy share from UPFDs varied markedly across the 22 European countries included, ranging from 14 to 44%, being the lowest in Italy and Romania, while the highest in the UK and Sweden. An overall modest decrease (2–15%) in UPFDs consumption is observed over time, except for Finland, Spain and the UK reporting increases (3–9%). Fine bakery wares and soft drinks were most frequently ranked as the main contributor. Countries with a higher sugar intake reported also a higher energy share from UPFDs, as most clearly observed for UPF (*r* = 0.57, *p* value = 0.032 for men; and *r* = 0.53, *p* value = 0.061 for women). No associations with fibre intake or high BMI were observed.

**Conclusion:**

Population-level UPFDs consumption substantially varied across Europe, although main contributors are similar. UPFDs consumption was not observed to be associated with country-level burden of high BMI, despite being related to a higher total sugar intake.

**Supplementary Information:**

The online version contains supplementary material available at 10.1007/s00394-021-02733-7.

## Introduction

Food processing, in particular the degree and purpose of the processing, is recognised to be an important determinant of food’s nutrient profile, and, therefore, diet quality and population’s health [1]. In public health nutrition, the NOVA classification system is regarded as an internationally recognised method for grouping foods according to the nature, extent, and purpose of the industrial processing [2]. The NOVA system classifies all foods and food products in four groups: (1) unprocessed or minimally processed foods, (2) processed culinary ingredients, (3) processed foods, and (4) ultra-processed foods and drinks (UPFDs) [3, 4]. As the Food Agriculture Organisation (FAO) states: ‘UPFDs are the formulation of ingredients, mostly of exclusive industrial use, typically created by series of industrial techniques processes’ [2]. Such ingredients and processes designed for the manufacture of UPFDs intend to extend their shelf life but also make them profitable, palatable, attractive and easy-to-consume [1, 2, 5]. However, these foods often have a suboptimal nutritional profile, are energy dense, i.e. they are low in fibre and micronutrients, but high in saturated fats, salt, and sugars inducing a high glycaemic load, hence best to avoid or minimise their consumption [1, 2, 5]. Through this suboptimal profile, diets rich in UPFDs may therefore increase the risk of elevated body mass index (BMI) and contribute to the burden of non-communicable diseases (NCDs) [6]. Emerging evidence from observational cross-sectional and cohort studies has identified positive associations between UPFDs consumption and at least one adverse NCD outcome [7, 8], such as high BMI [7], type 2 diabetes [9] as well as a higher risk of cardiovascular disease [10] and all-cause mortality [11].

Previous research using total food/drink volume sales per capita from Euromonitor including 80 countries showed that UPFD volume sales is the highest in Western Europe, North America, and Australasia, while also alarmingly increasing in other world regions [12]. Baked goods, including cakes, pastries, industrial breads, and soft drinks ranked among the top contributors to sales of UPFDs [12]. In Europe, UPFDs contributed to an average of 25% of the total dietary energy with the lowest estimates observed in Portugal and Italy and the highest in Germany and the UK, as estimated from the household budget survey data from 19 European countries [13]. Only a few studies from Belgium [14], France [15], Portugal [16], and the UK [17, 18] have used national representative individual-level dietary data reporting a total proportion of daily energy intake from UPFDs of 30, 31, 24, and 55%, respectively, with the highest share for baked goods and confectionary, processed meats, and soft drinks [13–15].

To date, a comprehensive overview of how much UPFDs contribute to the diets across all of Europe, as estimated from national individual-level dietary survey data, is lacking. This study aims to characterize European food consumption patterns in terms of their consumption of UPFDs, including the identification of the most consumed UPFDs and changes over time where possible, using aggregated national dietary survey data as available in the European Food Safety Authority (EFSA) Comprehensive European Food Consumption Database. In addition, the present study aims to analyse whether high consumption of UPFDs is associated with poorer dietary quality, as operationalised by the dietary intakes of fibre and sugar and the population burden of BMI.

## Materials and methods

### Food consumption data

Country-level publicly available dietary data estimated from individual-level dietary surveys were obtained from the Comprehensive European Food Consumption Database developed and maintained by the EFSA since 2011 [19]. Countries with national dietary survey data collected for at least 2 days by means of food records or 24-h dietary recalls were selected for the study. From the 25 European countries reporting survey dietary data to EFSA, the 22 countries for which adult food consumption data were available for at least 2 days were selected, excluding Bulgaria, Poland, and Slovakia [19]. The most recent survey year for each country was used to retrieve food consumption by sex, based on the individual mean consumption of the total survey period, although this year varied considerably between countries, i.e. from 2003 for Czech Republic and Hungary to 2017 for Slovenia. Food consumption data were collected by means of at least two 24-h recalls in 15 countries (i.e. Austria, Belgium, Croatia, Cyprus, Czech Republic, Estonia, Finland, France, Germany, Greece, Latvia, the Netherlands, Portugal, Slovenia, Spain), of which six of them were supplemented by either a food frequency questionnaire (i.e. Belgium and France), food propensity questionnaire (i.e. Latvia, Portugal and Slovenia) or lifestyle questionnaire covering supplement use and alcohol consumption (i.e. the Netherlands), and seven countries solely used food records as dietary assessment method (i.e. Denmark, Hungary, Ireland, Italy, Romania, Sweden, and the UK) with the number of days varying between 3 and 7 days.

Summary statistics of food consumption data reported in grams/day and presented according the sixth level of the ‘Exposure Hierarchy’ of the food classification and description system FoodEx2 [20] were retrieved for the adult population (aged 18–64 years). A number of 2287 different food items coded with a unique FoodEx2-code were consumed in the 22 European countries, and countries reporting on average 565 different food items (with a range from 289 to 995).

### Classification of foods according to the NOVA classification

The foods consumed across Europe and retrieved from EFSA, were classified as ‘unprocessed or minimally processed’, ‘processed culinary ingredients’, ‘processed’, and ‘ultra-processed’ according to the NOVA classification [3, 5, 21] (Supplementary Table 1). This classification relied on the coding details of FoodEx2, and on the level of food disaggregation when reporting food consumption. For example, composite dishes, fine bakery wares, doughs and pre-mixes, dairy-based desserts like puddings (whether home-prepared or not), that are included as such in FoodEx2, were not disaggregated into core ingredients, and were all classified as ultra-processed. Alcoholic beverages were kept as a separate group, as they were initially not included in the NOVA classification [2].

### Linkage with food composition databases

The collected food consumption data from EFSA, as coded by FoodEx2, was linked with detailed information on nutritional composition, using the Dutch Food Composition Database (NEVO) [22]. This linkage was initiated by the Dutch Food Consumption Survey 2012–2016 [23], where the foods consumed were coded by NEVO and FoodEx2. Foods reported by the European countries included in the present study were matched to the NEVO code that most closely resembled the sixth level of description of the FoodEx2 ‘Exposure Hierarchy’. This linkage with NEVO allowed the calculation of the dietary composition, and in particular the energy content of the diet for the calculation of the proportion of energy derived from UPFDs, as well as the fibre and sugar content of the diet, as two simple measures of carbohydrate intake and diet quality, also directly associated with body weight and BMI [24, 25].

### Countries burden of high BMI

Countries’ burden of high BMI was obtained from the latest release (2019) of the Global Burden of Disease (GBD) Study [26]. The GBD global health data exchange (GBDx) tool was used to extract age-standardised rates of Years Lived with Disability (YLD) (per 100,000) for high BMI, defined as BMI ≥ 25 kg/m^2^, for men and women separately and in the year matching the dietary survey, for each country [27]. This disability-adjusted measure, capturing incidence as well as duration of the disability and its associated disability weight, reflects the non-fatal burden of high BMI, hereby providing insight to the severity of living with that condition at the country level.

### Analysis

Using the sixth level of the FoodEx2 ‘Exposure Hierarchy’, the consumption of UPFDs was calculated, expressed as the proportion of daily food intake from UPFDs and the proportion of daily energy intake from UPFDs. The consumption of UPFDs was further segregated according to their physical form (solid vs liquid) into foods (UPFs) and drinks (UPDs), because of their possible implications for food choices, energy balance and body weight management [28]. UPFs included crackers and similar additional bread products, cereal bars, flakes and popped cereals, fine bakery wares, doughs and pre-mixes, nut spreads, fruit chips and chocolate coated dried fruit, meat specialities (meat spread/pate), sausages, fish specialities (fish paste), sweetened or flavoured dairy products, cheese spreads, manufactured eggs, sweetening ingredients (e.g. polyols) and table-top sweeteners, chocolate, candies and confectionary, sweet bars, spoonable water-/dairy-based desserts, margarines and other blended fats and oils, solid foods for particular diets and food supplements, meat imitates, composite dishes (including ready-to-eat meals and salads), seasoning, sauces and condiments, and other ingredients. UPDs included flavoured or sweetened milk and yoghurt drinks, milkshakes, fruit and vegetable juices (not 100% from named source), soft drinks, diet soft drinks, hot cocoa beverages, oral rehydration and carbohydrate-electrolyte solutions, milk imitates, and soups.

Subsequently, the mean contribution of the above-mentioned food subgroups to the dietary share of UPFDs by sex was calculated to identify the foods with the highest contribution to the consumption of UPFDs, with the top five of UPFs and the top three of UPDs identified for the UPFDs expressed in grams per day and in energy per day. For the 11 countries with recurrent dietary surveys over time, i.e. Austria, Belgium, Denmark, Finland, France, Latvia, Ireland, the Netherlands, Spain, Sweden, and the United Kingdom, we analysed their changes in UPFDs consumption, provided that the same dietary assessment method was used for dietary data collection, i.e. either the 24-h recall or food record for the recurrent dietary surveys.

Bivariate groupings were established by considering overlapping of the tertiles for measures of the dietary energy share of UPFDs, and measures of diet quality, represented by dietary fibre and total sugar intake, and of population burden of high BMI. Potential relationships were visualised in scatterplots, using colours to depict the different methods of dietary assessment, and were described using Spearman’ rank correlation coefficient (*r*) with *p* values adjusted for multiple testing according to Sidak, stratified by the physical form of the food (e.g. solid vs liquid) and sex. A two-sided *p* value below 0.05 was considered as statistically significant, and all analyses were conducted in STATA (Release SE 16.1/SE. College Station, TX: StataCorp LP).

## Results

### Consumption of ultra-processed foods and drinks

Table 1 shows for each country the total food consumption in grams per day and in energy per day, and the UPFD consumption as expressed in proportion of daily food intake and of energy intake, stratified by sex. The average consumption of UPFDs in adults across Europe was 328 g/day and the average proportion of daily food consumption amount from UPFDs was 12.0%. The average energy intake from UPFDs was 562 kcal/day, representing an average share of total energy intake of 27.2% (Table 1).

**Table 1:** General characteristics of the most recent dietary surveys conducted in the European adult population, as available by EFSA, and total daily consumption and ultra-processed foods and drinks, expressed in grams and in energy intakes, ordered alphabetically and stratified by sex

Country	Method of dietary assessment	Survey CODE	Year	Total daily food consumption				Ultra-processed foods and drinks consumption					
				Grams	% solid food	Energy	% EN solid food	% UPFD	% UPF	% UPD	%EN UPFD	% EN UPF	% EN UPD
Eur—22 countries	–	–	–	2734	39	2065	87	12.0	6.7	5.3	27.2	24.8	2.4
Men													
Eur—22 countries	–	–	–	2971	39	2415	86	12.8	7.0	5.8	26.4	24.1	2.3
Austria	24HR	AT-NATIONAL-2016	2014	4091	28	2496	87	13.4	6.6	6.8	31.7	28.1	3.6
Belgium	24HR, FFQ	NATIONAL-FCS-2014	2014	3075	35	2335	87	17.7	6.9	10.8	31.9	28.8	3.1
Croatia	24HR, 48HR	NIPNOP-HAH-2011-2012	2011	3039	36	2424	88	8.9	4.0	4.9	18.5	16.2	2.3
Cyprus	24HR	CY 2014-2017-LOT2	2014	3154	35	2255	89	8.0	3.7	4.3	20.3	18.0	2.3
Czech Republic	24HR	SISP04	2003	3504	33	3003	84	12.4	7.5	4.9	27.0	24.7	2.3
Denmark	Food record	DANSDA 2005-08	2005	3855	28	2630	79	9.5	3.8	5.7	25.3	21.2	4.1
Estonia	24HR	DIET-2014-EST-A	2013	3046	37	2215	83	6.7	3.9	2.7	17.4	16.0	1.4
Finland	48HR	FINDIET2012	2012	3416	33	2399	83	11.0	7.9	3.1	31.0	29.6	1.4
France	24HR, FFQ	INCA3	2014	3284	38	2553	89	11.4	7.3	4.1	28.4	26.5	1.9
Germany	24HR	NATIONAL NUTRITION SURVEY II	2007	3626	28	2527	81	16.6	8.8	7.8	38.0	34.5	3.5
Greece	24HR	GR-EFSA-LOT2 2014-2015	2014	1930	51	2235	89	11.0	5.9	5.1	20.1	19.0	1.1
Hungary	Food record	NATIONAL REPR SURV	2003	2056	57	2637	89	16.5	6.5	10.0	18.0	15.7	2.3
Ireland	Food record	NANS 2012	2008	3173	34	2684	78	13.2	8.3	4.9	31.8	29.7	2.1
Italy	Food record	INRAN SCAI 2005-06	2005	2275	51	2338	92	6.4	4.1	2.3	13.0	12.2	0.8
Latvia	24HR, FPQ	LATVIA_2014	2012	2546	49	2488	91	14.3	11.5	2.8	32.0	31.0	1.0
The Netherlands	24HR, lifestyle questionnaire	FCS2016_CORE	2012	3578	32	2714	84	20.0	9.0	11.0	37.0	33.0	4.0
Portugal	24HR, FPQ	IAN.AF 2015-2016	2015	2885	38	2230	85	11.2	4.1	7.1	19.8	16.1	3.7
Romania	Food record	DIETA PILOT ADULTS	2012	2255	59	2439	92	10.8	5.0	5.8	14.6	12.8	1.8
Slovenia	24HR, FPQ	SI.MENU-2018	2017	2566	42	2106	91	9.5	6.1	3.4	21.7	20.5	1.2
Spain	24HR	ENALIA2	2013	2148	40	1725	85	11.5	6.1	5.4	25.0	22.9	2.1
Sweden	Food record	RIKSMATEN 2010	2010	2825	40	2415	84	22.9	15.7	7.2	40.6	37.9	2.7
United Kingdom	Food record	NDNS ROLLING PROGRAMME YEARS 1-3	2008	3040	32	2270	81	18.3	10.4	7.9	39.7	36.6	3.1
Women													
Eur—22 countries	–	–	–	2538	38	1787	89	11.2	6.4	4.8	27.6	25.2	2.4
Austria	24HR	AT-NATIONAL-2016	2014	3467	27	1842	88	10.1	5.5	4.6	30.2	27.2	3.0
Belgium	24HR, FFQ	NATIONAL-FCS-2014	2014	2610	34	1676	90	14.4	6.3	8.1	31.7	29.1	2.7
Croatia	24HR, 48HR	NIPNOP-HAH-2011-2012	2011	2592	35	1749	91	7.7	3.3	4.4	19.7	17.1	2.6
Cyprus	24HR	CY 2014-2017-LOT2	2014	2361	33	1525	89	6.6	3.7	2.9	21.4	19.7	1.7
Czech Republic	24HR	SISP04	2003	2606	35	1880	91	11.0	6.1	4.9	28.2	25.5	2.7
Denmark	Food record	DANSDA 2005-08	2005	3519	27	1971	81	8.2	3.0	5.2	24.8	20.1	4.7
Estonia	24HR	DIET-2014-EST-A	2013	2519	37	1566	88	5.6	3.7	1.9	18.4	17.1	1.3
Finland	48HR	FINDIET2012	2012	3124	33	1894	87	9.3	7.2	2.1	32.5	31.2	1.3
France	24HR, FFQ	INCA3	2014	2856	37	1983	90	10.3	7.0	3.3	29.1	27.3	1.8
Germany	24HR	NATIONAL NUTRITION SURVEY II	2007	3289	27	1882	84	13.2	7.9	5.3	38.9	35.5	3.4
Greece	24HR	GR-EFSA-LOT2 2014-2015	2014	1602	53	1694	92	10.0	6.3	3.7	23.7	22.7	1.0
Hungary	Food record	NATIONAL REPR SURV	2003	1702	59	2049	91	13.8	6.1	7.7	17.1	15.0	2.1
Ireland	Food record	NANS 2012	2008	2533	34	1904	83	13.4	8.3	5.1	35.3	32.9	2.4
Italy	Food record	INRAN SCAI 2005-06	2005	2087	50	1909	94	6.1	4.2	1.9	13.8	13.0	0.8
Latvia	24HR, FPQ	LATVIA_2014	2012	2192	44	1728	92	10.3	8.6	1.7	30.7	29.9	0.8
The Netherlands	24HR, lifestyle questionnaire	FCS2016_CORE	2012	3217	29	1964	86	16.6	7.3	9.3	37.3	32.9	4.4
Portugal	24HR, FPQ	IAN.AF 2015-2016	2015	2350	36	1593	88	11.0	4.8	6.2	24.5	20.7	3.8
Romania	Food record	DIETA PILOT ADULTS	2012	1919	61	1976	94	11.5	4.9	6.6	15.9	13.6	2.3
Slovenia	24HR, FPQ	SI.MENU-2018	2017	2303	38	1520	93	7.5	5.4	2.1	23.5	22.3	1.2
Spain	24HR	ENALIA2	2013	1878	40	1410	86	11.4	5.9	5.5	25.3	23.0	2.3
Sweden	Food record	RIKSMATEN 2010	2010	2585	37	1909	86	20.9	14.4	6.5	43.8	40.8	3.0
United Kingdom	Food record	NDNS ROLLING PROGRAMME YEARS 1-3	2008	2515	33	1698	85	17.2	9.8	7.4	41.3	38.0	3.3

*24HR* 24-h dietary recall, *48HR* 48-h dietary recall, *%EN* percentage of energy intake, i.e. amount of kcal relative to total amount of kcal consumed, *Eur* Europe, *FFQ* Food Frequency Questionnaire, *FPQ* Food Propensity Questionnaire, *UPD* ultra-processed drinks, *UPF* ultra-processed foods, *UPFD* ultra-processed foods and drinks

The proportion of daily food consumption amount deriving from UPFDs for men ranged from to 6.4% (Italy) and 6.7% (Estonia) to 20.0% (the Netherlands) and 22.9% (Sweden), of which on average around 55% was coming from UPFs. The consumption amount of UPDs was, however, higher than that of UPFs in Belgium, Denmark, Hungary, the Netherlands, Portugal and Romania (2–4% higher) and similar in Austria, Croatia, Cyprus, Estonia, Germany, Greece, Italy, Romania, and Spain. The share of dietary energy coming from UPFDs for men ranged from 12.9% (Italy) and 14.6% (Romania) to 39.7% (the UK) and 40.6% (Sweden), with on average around 90% of the dietary energy from UPFDs was coming from UPFs.

Similarly, for women, proportion of daily food consumption amount from UPFDs was low for Estonia (5.7%) and Italy (6.1%), and high for the Netherlands (16.6%), the United Kingdom (17.2%), and Sweden (20.9%), while the consumption amount of UPDs was similar to that of UPFs in Austria, Belgium, Croatia, Cyprus, Czech Republic, Denmark, Estonia, Hungary, Italy, the Netherlands, Portugal, Romania, and Spain. The share of dietary energy coming from UPFDs for women was also low in Italy (13.8%) and Romania (15.8%), while high in the UK (41.3%) and Sweden (43.8%).

### Top five of the ultra-processed foods

Overall, the most consumed UPFs among adults, both men and women, across Europe were fine bakery wares, when expressed in dietary energy share from UPFs (Table 2) and when expressed in daily food consumption amounts, i.e. grams per day (Supplementary Table 2).

**Table 2:** The top five ultra-processed foods consumed by European adults, as available by EFSA, ordered alphabetically and stratified by sex, expressed as the percentage of daily energy from ultra-processed foods

	Top five				
	1	2	3	4	5
Eur—22 countries	Fine bakery wares (26.9%)	Sausages (12.5%)	Composite dishes (9.5%)	Margarines (7.9%)	Sauces (7.6%)
Men					
Eur—22 countries	Fine bakery wares (25.2%)	Sausages (15.5%)	Composite dishes (9.7%)	Margarines (8.1%)	Sauces (7.9%)
Austria	Fine bakery wares (37%)	Sausages (11%)	Sauces (10%)	Chocolate (8%)	Composite dishes (7%)
Belgium	Fine bakery wares (24%)	Sauces (15%)	Chocolate (10%)	Margarines (10%)	Sausages (9%)
Croatia	Sausages (41%)	Fine bakery wares (13%)	Chocolate (7%)	Crackers and additional bread products (6%)	Margarines (5%)
Cyprus	Fine bakery wares (37%)	Crackers and additional bread products (18%)	Breakfast cereals (7%)	Composite dishes (5%)	Sauces (4%)
Czech Republic	Fine bakery wares (30%)	Sausages (28%)	Composite dishes (12%)	Margarines (4%)	Crackers and additional bread products (4%)
Denmark	Margarines (30%)	Sausages (10%)	Chocolate (9%)	Fine bakery wares (7%)	Sauces (6%)
Estonia	Sausages (28%)	Fine bakery wares (17%)	Margarines (9%)	Chocolate (8%)	Sauces (8%)
Finland	Margarines (27%)	Fine bakery wares (18%)	Sauces (10%)	Sausages (10%)	Sweetened/flavoured dairy products (9%)
France	Fine bakery wares (38%)	Sauces (13%)	Sausages (9%)	Crackers and additional bread products (8%)	Sweetened/flavoured dairy products (8%)
Germany	Fine bakery wares (23%)	Sausages (15%)	Composite dishes (13%)	Sauces (11%)	Margarines (9%)
Greece	Fine bakery wares (39%)	Crackers and additional bread products (20%)	Sauces (11%)	Margarines (5%)	Breakfast cereals (4%)
Hungary	Sausages (39%)	Margarines (16%)	Fine bakery wares (9%)	Chocolate (6%)	Crackers and additional bread products (5%)
Ireland	Fine bakery wares (22%)	Margarines (14%)	Composite dishes (12%)	Sauces (12%)	Breakfast cereals (10%)
Italy	Fine bakery wares (44%)	Sausages (17%)	Composite dishes (8%)	Water-/dairy-based desserts (7%)	Crackers and additional bread products (5%)
Latvia	Fine bakery wares (31%)	Sausages (17%)	Composite dishes (17%)	Sauces (16%)	Chocolate (6%)
The Netherlands	Fine bakery wares (18%)	Composite dishes (17%)	Sauces (12%)	Margarines (12%)	Chocolate (7%)
Portugal	Fine bakery wares (37%)	Sausages (8%)	Composite dishes (8%)	Breakfast cereals (6%)	Water-/dairy-based desserts (5%)
Romania	Sausages (27%)	Composite dishes (17%)	Fine bakery wares (11%)	Crackers and additional bread products (10%)	Margarines (7%)
Slovenia	Fine bakery wares (36%)	Sausages (23%)	Crackers and additional bread products (7%)	Sauces (6%)	Chocolate (6%)
Spain	Fine bakery wares (36%)	Crackers and additional bread products (15%)	Sausages (12%)	Sweetened/flavoured dairy products (7%)	Sauces (6%)
Sweden	Composite dishes (42%)	Fine bakery wares (14%)	Sauces (10%)	Margarines (7%)	Sausages (6%)
United Kingdom	Composite dishes (32%)	Fine bakery wares (15%)	Sauces (11%)	Margarines (8%)	Sausages (8%)
Women					
Eur—22 countries	Fine bakery wares (28.2%)	Sausages (9.5%)	Composite dishes (9.1%)	Margarines (9.1%)	Sauces (7.2%)
Austria	Fine bakery wares (42%)	Sauces (11%)	Chocolate (10%)	Composite dishes (6%)	Sausages (6%)
Belgium	Fine bakery wares (29%)	Sauces (13%)	Chocolate (10%)	Margarines (9%)	Sausages (7%)
Croatia	Sausages (23%)	Fine bakery wares (16%)	Chocolate (9%)	Margarines (9%)	Crackers and additional bread products (8%)
Cyprus	Fine bakery wares (40%)	Breakfast cereals (11%)	Crackers and additional bread products (11%)	Composite dishes (6%)	Chocolate (5%)
Czech Republic	Fine bakery wares (36%)	Sausages (13%)	Composite dishes (12%)	Margarines (6%)	Crackers and additional bread products (5%)
Denmark	Margarines (28%)	Chocolate (10%)	Fine bakery wares (7%)	Candies, confectionary (6%)	Sausages (6%)
Estonia	Fine bakery wares (23%)	Sausages (17%)	Chocolate (11%)	Margarines (8%)	Sauces (7%)
Finland	Margarines (26%)	Fine bakery wares (20%)	Sweetened/flavoured dairy products (11%)	Sauces (7%)	Chocolate (6%)
France	Fine bakery wares (40%)	Sauces (12%)	Sweetened/flavoured dairy products (9%)	Crackers and additional bread products (8%)	Sausages (6%)
Germany	Fine bakery wares (27%)	Composite dishes (15%)	Sauces (12%)	Sausages (9%)	Chocolate (6%)
Greece	Fine bakery wares (39%)	Crackers and additional bread products (25%)	Breakfast cereals (7%)	Margarines (6%)	Sauces (5%)
Hungary	Sausages (25%)	Margarines (21%)	Fine bakery wares (13%)	Crackers and additional bread products (6%)	Chocolate (6%)
Ireland	Fine bakery wares (25%)	Margarines (12%)	Composite dishes (12%)	Sauces (11%)	Breakfast cereals (9%)
Italy	Fine bakery wares (44%)	Sausages (11%)	Composite dishes (8%)	Water-/dairy-based desserts (7%)	Crackers and additional bread products (7%)
Latvia	Fine bakery wares (35%)	Sauces (14%)	Composite dishes (13%)	Chocolate (11%)	Sausages (10%)
The Netherlands	Fine bakery wares (20%)	Composite dishes (14%)	Sauces (11%)	Margarines (11%)	Chocolate (7%)
Portugal	Fine bakery wares (40%)	Composite dishes (9%)	Breakfast cereals (7%)	Water-/dairy-based desserts (5%)	Sausages (5%)
Romania	Sausages (18%)	Composite dishes (15%)	Crackers and additional bread products (14%)	Fine bakery wares (12%)	Margarines (7%)
Slovenia	Fine bakery wares (45%)	Sausages (10%)	Crackers and additional bread products (10%)	Breakfast cereals (5%)	Sauces (5%)
Spain	Fine bakery wares (35%)	Crackers and additional bread products (17%)	Sausages (9%)	Sweetened/flavoured dairy products (7%)	Sauces (5%)
Sweden	Composite dishes (41%)	Fine bakery wares (15%)	Sauces (10%)	Margarines (6%)	Chocolate (5%)
United Kingdom	Composite dishes (29%)	Fine bakery wares (16%)	Sauces (13%)	Margarines (7%)	Breakfast cereals (7%)

*Eur* Europe

The UPFs most frequently ranked among the top five contributors to the dietary energy share of UPFs were: fine bakery wares, sausages, sauces, margarines and composite dishes, followed by crackers and additional bread products, chocolate, breakfast cereals, chocolate, and least frequently ranked were sweetened or flavoured dairy products, water-/dairy-based desserts, and candies and confectionary (Table 2). From this list, fine bakery wares were ranked first in most countries (14 countries for men; 15 countries for women), followed by sausages (4 countries for men and 3 countries for women), and margarines and composite dishes (2 countries each for both men and women). Comparing men and women, the share of fine bakery wares was lower in men (25.2% vs 28.2%), while that of sausages was higher (15.5% vs 9.5%).

The top five of the UPFs consumed by European adults was slightly different when expressing in percentage of daily food consumption amounts as compared to dietary energy shares (Supplementary Table 2). Still, the largest contributor was the group of fine bakery wares (in 9 countries for men, and in 12 countries for women), but closely followed by composite dishes (in 6 countries for men, and in 5 countries for women), and after that by sausages (in 5 countries in men, and in 2 countries in women), and margarines (Denmark in both men and women), and also sweetened or flavoured dairy products (Finland for men and Finland and Estonia for women). Besides, these sweetened or flavoured dairy products were also identified as the top five contributor in the food consumption share of UPFs in 13 countries for men and 18 countries for women (while only in Finland, France and Spain when considering dietary energy share), most often at the expense of chocolate or margarine.

### Top three of ultra-processed drinks

Overall, the most consumed UPDs among adults across Europe were soft drinks, when expressed both in dietary energy share from UPDs (Table 3), or in daily food consumption amounts (Supplementary Table 3).

**Table 3:** The top three of the ultra-processed drinks consumed by European adults, as available by EFSA, ordered alphabetically and stratified by sex, expressed in percentage of daily energy intake from ultra-processed drinks

	Top three		
	1	2	3
Eur—22 countries	Soft drinks (5.0%)	Fruit/vegetables juices not 100% from named source (1.4%)	Sweetened/flavoured milk (1.1%)
Men			
Eur—22 countries	Soft drinks (5.7%)	Fruit/vegetables juices not 100% from named source (1.3%)	Sweetened/flavoured milk (0.9%)
Austria	Soft drinks (9%)	Fruit/vegetables juices not 100% from named source (2%)	Sweetened/flavoured milk (1%)
Belgium	Soft drinks (8%)	Sweetened/flavoured milk (1%)	Milk imitates (0.4%)
Croatia	Soft drinks (6%)	Fruit/vegetables juices not 100% from named source (6%)	Cocoa beverages (0.3%)
Cyprus	Soft drinks (10%)	Fruit/vegetables juices not 100% from named source (1%)	Soups (0.4%)
Czech Republic	Soft drinks (7%)	Fruit/vegetables juices not 100% from named source (1%)	Cocoa beverages (0.3%)
Denmark	Soft drinks (12%)	Fruit/vegetables juices not 100% from named source (2%)	Sweetened/flavoured milk (2%)
Estonia	Soft drinks (4%)	Fruit/vegetables juices not 100% from named source (2%)	Sweetened/flavoured milk (1%)
Finland	Soft drinks (2%)	Cocoa beverages (1%)	Sweetened/flavoured milk (0.5%)
France	Soft drinks (4%)	Cocoa beverages (1%)	Fruit/vegetables juices not 100% from named source (1%)
Germany	Soft drinks (4%)	Soups (3%)	Fruit/vegetables juices not 100% from named source (2%)
Greece	Soft drinks (3%)	Fruit/vegetables juices not 100% from named source (2%)	Sweetened/flavoured milk (0.2%)
Hungary	Soft drinks (6%)	Sweetened/flavoured milk (6%)	Cocoa beverages (1%)
Ireland	Soft drinks (5%)	Fruit/vegetables juices not 100% from named source (1%)	Sweetened/flavoured milk (0.4%)
Italy	Soft drinks (3%)	Fruit/vegetables juices not 100% from named source (3%)	Cocoa beverages (1%)
Latvia	Soft drinks (2%)	Fruit/vegetables juices not 100% from named source (1%)	Cocoa beverages (0%)
The Netherlands	Soft drinks (6%)	Sweetened/flavoured milk (2%)	Fruit/vegetables juices not 100% from named source (1%)
Portugal	Soft drinks (10%)	Sweetened/flavoured milk (4%)	Fruit/vegetables juices not 100% from named source (3%)
Romania	Soft drinks (8%)	Soups (4%)	Cocoa beverages (0.5%)
Slovenia	Soft drinks (3%)	Cocoa beverages (1%)	Fruit/vegetables juices not 100% from named source (1%)
Spain	Soft drinks (4%)	Milk imitates (1%)	Cocoa beverages (1%)
Sweden	Soft drinks (3%)	Soups (2%)	Fruit/vegetables juices not 100% from named source (1%)
United Kingdom	Soft drinks (5%)	Soups (1%)	Milkshakes (0.4%)
Women			
Eur—22 countries	Soft drinks (4.5%)	Fruit/vegetables juices not 100% from named source (1.5%)	Sweetened/flavoured milk (1.3%)
Austria	Soft drinks (6%)	Fruit/vegetables juices not 100% from named source (2%)	Sweetened/flavoured milk (1%)
Belgium	Soft drinks (6%)	Sweetened/flavoured milk (1%)	Milk imitates (1%)
Croatia	Fruit/vegetables juices not 100% from named source (6%)	Soft drinks (6%)	Cocoa beverages (1%)
Cyprus	Soft drinks (6%)	Fruit/vegetables juices not 100% from named source (1%)	Soups (1%)
Czech Republic	Soft drinks (7%)	Fruit/vegetables juices not 100% from named source (3%)	Cocoa beverages (0.3%)
Denmark	Soft drinks (14%)	Sweetened/flavoured milk (3%)	Fruit/vegetables juices not 100% from named source (2%)
Estonia	Fruit/vegetables juices not 100% from named source (2%)	Soft drinks (2%)	Sweetened/flavoured milk (2%)
Finland	Cocoa beverages (1%)	Soft drinks (1%)	Sweetened/flavoured milk (1%)
France	Soft drinks (4%)	Cocoa beverages (1%)	Fruit/vegetables juices not 100% from named source (1%)
Germany	Soups (3%)	Soft drinks (2%)	Fruit/vegetables juices not 100% from named source (2%)
Greece	Soft drinks (2%)	Fruit/vegetables juices not 100% from named source (1%)	Cocoa beverages (0.3%)
Hungary	Sweetened/flavoured milk (7%)	Soft drinks (4%)	Cocoa beverages (1%)
Ireland	Soft drinks (4%)	Fruit/vegetables juices not 100% from named source (1%)	Sweetened/flavoured milk (0.5%)
Italy	Fruit/vegetables juices not 100% from named source (3%)	Soft drinks (2%)	Cocoa beverages (1%)
Latvia	Soft drinks (1%)	Fruit/vegetables juices not 100% from named source (1%)	Cocoa beverages (0.2%)
The Netherlands	Soft drinks (5%)	Sweetened/flavoured milk (3%)	Fruit/vegetables juices not 100% from named source (2%)
Portugal	Sweetened/flavoured milk (6%)	Soft drinks (5%)	Fruit/vegetables juices not 100% from named source (2%)
Romania	Soft drinks (8%)	Soups (5%)	Cocoa beverages (1%)
Slovenia	Soft drinks (2%)	Sweetened/flavoured milk (1%)	Fruit/vegetables juices not 100% from named source (1%)
Spain	Soft drinks (4%)	Milk imitates (2%)	Cocoa beverages (1%)
Sweden	Soft drinks (2%)	Soups (2%)	Fruit/vegetables juices not 100% from named source (1%)
United Kingdom	Soft drinks (5%)	Soups (2%)	Cocoa beverages (0.4%)

*Eur* Europe

Soft drinks ranked as the main contributor to the dietary energy share of UPDs for men in all European countries included, while for women also fruit juices not 100% from named source (3 countries), sweetened or flavoured milk (2 countries), cocoa beverages and soups (1 country each) were identified as main contributors (Table 3). Expressed in daily food consumption amounts, soft drinks consistently ranked first in all countries for both men and women (Supplementary Table 3). In addition, diet soft drinks were observed in the top three of UPDs in nine countries for both men and women.

### Time-changes in ultra-processed foods and drinks

**Fig. 1:**
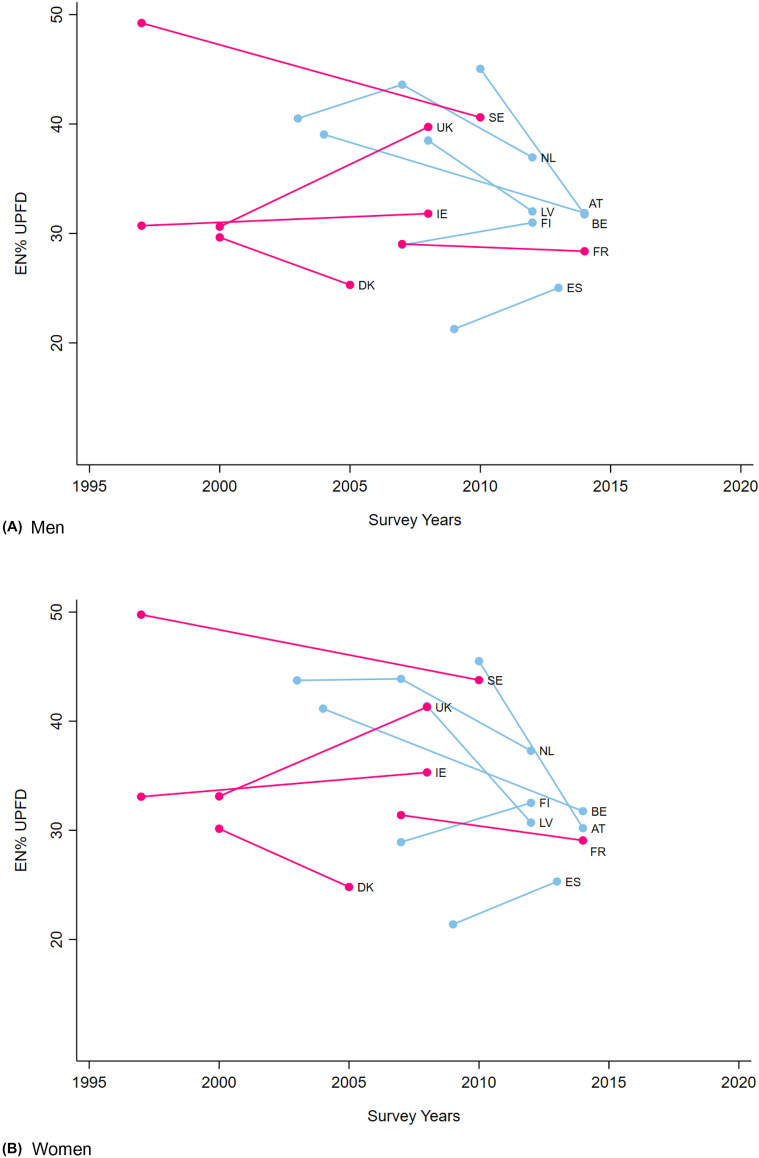
Time-changes of the dietary energy share from ultra-processed foods and drinks in the European adult population, with recurrent dietary survey intake data available by EFSA, stratified by sex and method of dietary assessment. Legend:  24-h recalls;  Food records. *AT* Austria, *BE* Belgium, *DK* Denmark, *EN%* energy percentages, *ES* Spain, *FI* Finland, *FR* France, *LV* Latvia, *IE* Ireland, *NL* the Netherlands, *SE* Sweden, *UK* United Kingdom, *UPFD* ultra-processed foods and drinks

Figure 1 shows the time-changes in the dietary energy share from UPFDs for countries with available data. In six countries, a decrease in the share of dietary energy from UPFDs was observed, with generally a slightly larger decrease for women than for men, and the largest decrease for Austria (13% for men vs 15% for women), Belgium (7% vs 9%), and Latvia (6% vs 11%). No clear time-changes were observed for Ireland and France, and Finnish men (less than 2%), while the share of dietary energy from UPFDs was higher for the most recent survey in the UK (9% and 8% for men and women, respectively), Spain (4% for both men and women) and Finland (4% for women only).

### Are countries with a higher consumption of ultra-processed foods and drinks associates of a lower consumption of dietary fibre and a higher consumption of total sugar intake?

**Fig. 2:**
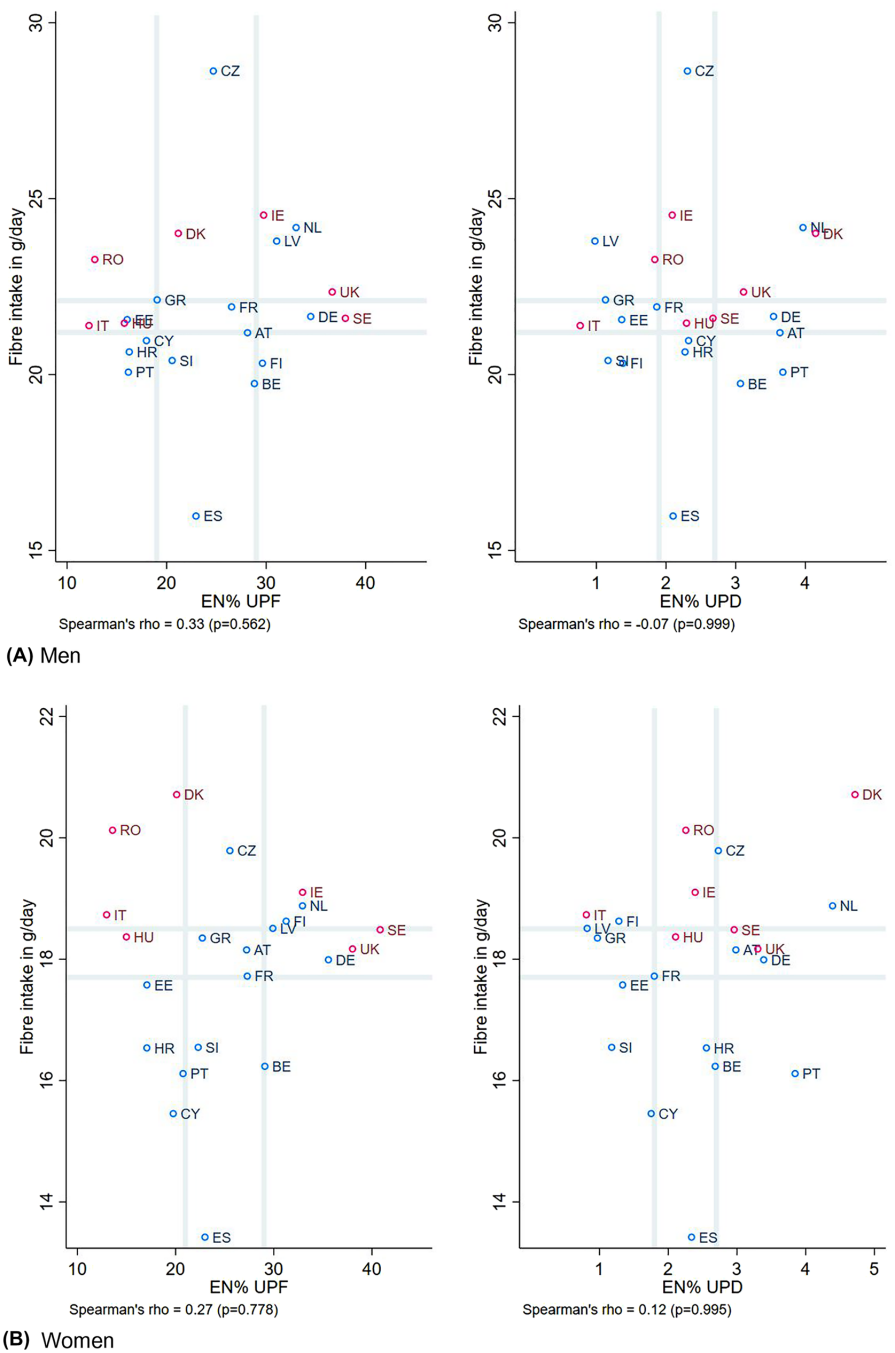
Scatterplot of fibre intake (in grams per day) against the percentage of energy coming from ultra-processed foods and drinks, stratified sex, and dietary assessment method. Legend:  24-hR; Food records. Grey vertical and horizontal gridlines indicate tertiles dividing lines for the measures: the share of dietary energy from UPFs (at 19.0 and 28.8 EN% for men, and at 20.7 and 29.1 EN% for women) and UPDs (at 1.9 and 2.7 EN% for men, and at 1.8 and 2.7 EN% for women), and fibre intake (at 21.2 and 22.1 g/day for men, and at 17.7 and 18.5 g/day for women). *AT* Austria, *BE* Belgium, *CY* Cyprus, *CZ* Czech Republic, *DE* Germany, *DK* Denmark, *EE* Estonia, *EN%* energy percentage, *ES* Spain, *FI* Finland, *FR* France, *GR* Greece, *HR* Croatia, *HU* Hungary, *IE* Ireland, *IT* Italy, *LV* Latvia, *NL* the Netherlands, *PT* Portugal, *RO* Romania, *SE* Sweden, *SI* Slovenia, *UK* United Kingdom, *UPD* ultra-processed drinks, *UPF* ultra-processed foods

**Fig. 3:**
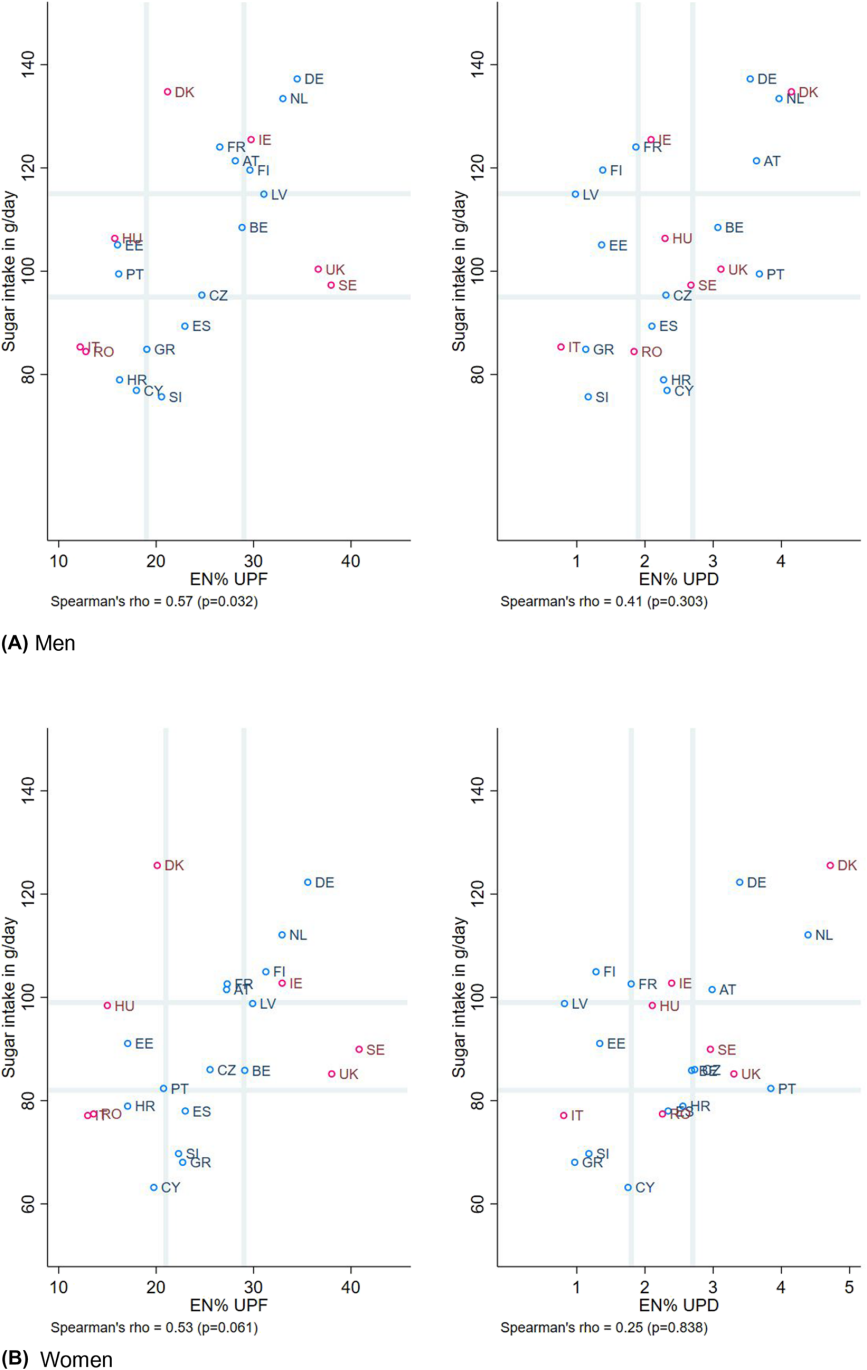
Scatterplot of total sugar intake (in grams per day) against the percentage of energy coming from ultra-processed foods and drinks, stratified by sex, and dietary assessment method. Legend:  24-hR;  Food records. Grey vertical and horizontal gridlines indicate tertiles dividing lines for the measures: the share of dietary energy from UPFs (at 19.0 and 28.8 EN% for men, and at 20.7 and 29.1 EN% for women) and UPDs (at 1.9 and 2.7 EN% for men, and at 1.8 and 2.7 EN% for women), and sugar intake (at 95.4 and 115.0 g/day for men, and at 82.4 and 98.8 g/day for women). *AT* Austria, *BE* Belgium, *CY* Cyprus, *CZ* Czech Republic, *DE* Germany, *DK* Denmark, *EE* Estonia, *EN%* energy percentage, *ES* Spain, *FI* Finland, *FR* France, *GR* Greece, *HR* Croatia, *HU* Hungary, *IE* Ireland, *IT* Italy, *LV* Latvia, *NL* the Netherlands, *PT* Portugal, *RO* Romania, *SE* Sweden, *SI* Slovenia, *UK* United Kingdom, *UPD* ultra-processed drinks, *UPF* ultra-processed foods

No associations were found for consumption of UPFDs and fibre intake (Fig. 2), while countries with diets high in total sugar intake were also the countries with a higher share of dietary energy from UPFs and UPDs, as seen for both men and women (Fig. 3). Weak-moderate correlations were only seen for the energy percentage of UPFs and total sugar intake (*r* = 0.57, *p* value = 0.032 for men; and *r* = 0.53, *p* value = 0.061 for women).

### Do countries with a higher consumption of ultra-processed foods and drinks also have a higher burden of high BMI?

**Fig. 4:**
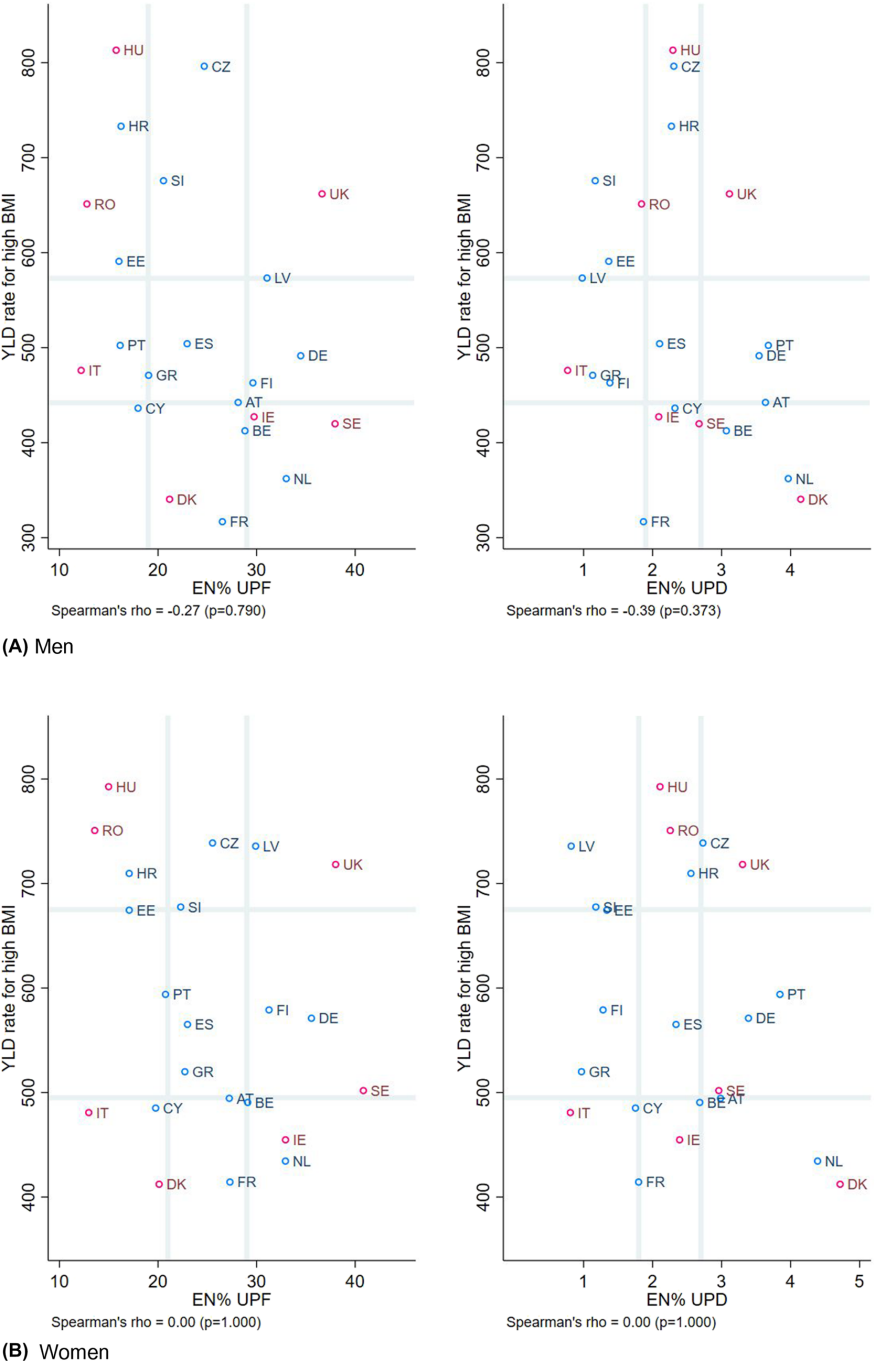
Scatterplot of YLD rate for high BMI against the percentage of energy derived from ultra-processed foods and drinks, stratified by sex, and dietary assessment method. Legend:  24-hR;  Food records. Grey vertical and horizontal gridlines indicate tertiles dividing lines for the measures: the share of dietary energy from UPFs (at 19.0 and 28.8 EN% for men, and at 20.7 and 29.1 EN% for women) and UPDs (at 1.9 and 2.7 EN% for men, and at 1.8 and 2.7 EN% for women), and YLD for high BMI per 100,000 (at 443 and 573 for men, and at 495 and 675 for women). *AT* Austria, *BE* Belgium, *CY* Cyprus, *CZ* Czech Republic, *DE* Germany, *DK* Denmark, *EE* Estonia, *EN%* energy percentage, *ES* Spain, *FI* Finland, *FR* France, *GR* Greece, *HR* Croatia, *HU* Hungary, *IE* Ireland, *IT* Italy, *LV* Latvia, *NL* the Netherlands, *PT* Portugal, *RO* Romania, *SE* Sweden, *SI* Slovenia, *UK* United Kingdom, *UPD* ultra-processed drinks, *UPF* ultra-processed foods, *YLD* Years Lived with Disability, in age-standardised rates per 100,000

Figure 4 shows the bivariate analyses for the potential association for the burden of high BMI and energy percentage of UPFDs, stratified by sex and dietary assessment method, as visualised by scatterplots. Countries’ burden of high BMI was not observed to increase across increasing percentages of energy from UPFDs, and no correlations were observed.

## Discussion

Using data on country-specific dietary intakes, the present study found that the share of dietary energy originating from UPFDs consumption varied markedly across the 22 European countries included in the analysis, ranging from 14 to 44% with the lowest share for Italy, Romania and Hungary, and the highest for the Netherlands, Germany, the UK and Sweden. Fine bakery wares and soft drinks were the main contributor to the dietary energy share of UPFs and UPDs, respectively.

Our results on the dietary energy share of UPFDs are in the range as those reported earlier using food purchase data across Europe [13]. However, the levels observed in the present study differed from those reported in previous studies that also used individual-level national dietary data analysed at the individual level. Our estimates were slightly higher for Belgium [14], but slightly lower for France [15] and Portugal [16] and up to around 20% lower for the UK for surveys (years) similar to the ones included in the present analyses, and also much lower as compared to earliest results (dated from 1995 to 2000) from middle-aged adults from ten European countries participating in the European Prospective Investigation into Cancer and Nutrition (EPIC) that reported a range between 60 and 80% [30]. These discrepancies may be due to differential use of the NOVA classification and its application depending on the variety of foods captured in the dietary database selected for the estimation of UPFDs [31, 32]. In our study, the use of FoodEx2 did not allow to disentangle breads withhout extra ingredients, except nuts and seeds, that are processed, from the industrially produced ones, that are ultra-processed, contrary to what is done in reports from individual countries where information on production and/or packaging is available [15, 16, 18], hence likely resulting in an underestimation in our study. The exclusion of distilled alcoholic beverages from our definition of UPFDs might also marginally underestimate our results, since the average share of total energy intake from these beverages was 0.42% (with a range from 0 to 2.5%) for the European countries included, representing when included in UPFDs an average contribution of 1.6 percent (data not shown).

Comprehensive understanding of the contribution of UPFDs to dietary quality would require describing consumption in amounts of grams and energy intakes, rather than the dietary energy share of UPFDs alone, as often done in the literature. This is clearly illustrated by the contribution of drinks to UPFDs consumption that is between 16 and 64% of the total UPFDs amount consumed and only between 3 and 19% of the energy intake from UPFDs. Therefore, describing food consumption solely in energy percentages functionally disregards the role of low-/non-energy bearing foods, such as diet soft drinks, on dietary quality in terms of the extent and purpose of industrial processing, as assessed by the NOVA classification.

Most prior studies found that higher consumption of UPFDs were associated with a higher BMI, as reported for example by a recent narrative review covering study designs from three ecological, ten cross-sectional and two prospective cohort studies, and one randomised controlled crossover trial [7]. Our results, however, do not fully support these previous observations. The ecological design of our analysis is mostly relevant for a descriptive comparison across Europe and does not account for socio-demographic drivers of food choices, such as socio-economic status, or other determinants of high BMI, such as physical inactivity. Thus, with population being the unit of analysis, caution must be applied, as findings cannot be extrapolated to high-risk individuals within countries because of ecological fallacy. Nevertheless, the consumption of UPFDs that are often of low-nutritional quality (i.e. energy-dense, low-fibre, nutrient-depleted, and/or high-sodium, and high-added sugars) would in principle hinder adherence to a healthy, optimal, diet. Relating to dietary quality of the foods consumed, it is not surprising that the intake of total sugars is higher with increasing intakes of UPFDs [18, 29, 33–35] because they are among the major sources of added sugar in the diet [5]. Our study could confirm also this tendency for total sugars, but not the anticipated trend in the opposite direction for dietary fibre, i.e. a nutrient closely linked with unprocessed or minimally processed foods, like whole grains, fruits, legumes and vegetables. Yet, this null-finding emphasises that UPFDs might be as crucial in nutrient provision as unprocessed or minimally processed foods [36, 37], also related to the fact that UPFDs account for a large part of European diets and contributes on average one-sixth of total daily dietary fibre intake (data not shown). Moreover, at the food level, when compared to the foods not ultra-processed, the UPFDs often contain more carbohydrates of low-quality, reflected utmost in the levels of added sugar (3.4% for foods not ultra-processed vs 19.3% for UPFDs) rather than dietary fibre (0.87 g/100 kcal vs 0.67 g/100 kcal), as reported by the nutrient profiles of the foods consumed by USA youths [38].

As expected, the share of dietary energy from UPFDs across Europe is lower than those reported for other Western countries such as the USA [33, 39, 40] and Canada [35], reporting intakes of up to around 60% of the energy intake. Still, our study confirms the existence of a geographical gradient for the contribution of UPFDs to European diets, i.e. they are highly dominating the diets of Western European countries, but not yet those of countries located in Central, the East and the South of Europe, consistent with prior studies (one dated from 1995 to 2000 [30] and two using purchase data [12, 13]).

Sales of UPFDs have on average slightly decreased over the period 2002–2016 for Western Europe, notwithstanding remaining high, while those of Central and Eastern Europe increased reflecting the move towards more processed diets [12]. Comparison of time-changes in UPFDs sales of individual European countries and our observations for the countries with recurrent food consumption surveys showed disagreements, pinpointing the challenges involved in the different methods of dietary data collection and analyses [41]. However, sales and purchase data for food and drinks can only be regarded as a proxy of food consumption data, because of the food practices between purchase and final consumption, including food preparation, distribution among household members and waste and/or in the accuracy of reporting dietary data.

The present study provides further support for the utilization of comprehensive nutritional databases in epidemiological studies to address current dietary concerns as well as the diet-health relationships. Cross-country comparison of dietary data, collected at the individual-level, is, however, challenged by the conduct of the dietary surveys related to the survey characteristics and data collection methods that may influence comparability of the results. To overcome this limitation in the future, the EFSA has since 2014 launched European Union Menu Project, i.e. an Europe-wide initiative aimed at a standardised collection of accurate, harmonised and detailed food consumption data across Member States [42]. For the time being, the present analyses were based on dietary data collected by means of at least two days of food records or 24-h recalls, since both are reporting on the foods and amounts that are actually consumed by an individual on specific days, hereby allowing greater specificity for describing foods, although its limitations of being resource-intensive, and subject to misreporting [43].

Expressing food consumption data as energy percentages might potentially partly account for the extraneous variation in dietary estimates due to measurement error. However, the use of the Dutch food composition database NEVO to calculate dietary composition, including the intake of energy, fibre and sugar, of the different national diets across Europe might limit the accuracy of our estimates, and decrease variability across countries. This is because of the lack of data to account for country-specific food composition, but also the foods and composite dishes available in the different countries are not necessarily produced/prepared in the same manner despite globalisation of dietary patterns. Nevertheless, the use of the same food composition database to estimate dietary composition increases cross-country comparability by cancelling out any potential systematic bias that may exist in country-specific databases and likely to vary in magnitude and direction. Our analyses, therefore, allow for direct comparison of the nutrient intakes, as any differences in nutrient intakes exclusively originate from the composition of diet instead of a mingling of food and dietary composition.

Another attempt for dietary data harmonisation of this study related to the use of a common food classification system, i.e. FoodEx2, to report food consumption consistently across countries, and subsequently calculate intakes according to the NOVA classification and food subgroups uniformly. Still, results would be highly relying on the coding-details of FoodEx2, and in all probability influence our estimate of UPFDs consumption, as previously discussed. Any possible discrepancy with previous reports may be due to the report of alcoholic beverages that were not classified and/or the composite dishes that were all classified as ultra-processed without considering any classification according to the NOVA classification for the disaggregated ingredients of home-prepared dishes, as often done when individual-level data are available. The present study applying the EFSA nutritional database constructed from individual-level dietary data highlights the importance of using a common food classification system and the same food composition database for aligning dietary data from the consumer domain, and hereby enabling cross-country comparisons of the diet, from the consumption of foods to the intake of nutrients.

## Conclusion

This study aimed at describing UPFDs consumption across Europe found considerable variation in the proportion of amounts consumed according to energy intakes by European adults, irrespective of sex, while similar main contributors to the UPFDs consumption were identified across countries and sex. Population-level consumption of UPFDs did not appear to be associated with a country-level burden of high BMI, despite being related to higher total sugar intake.

## Supplementary Information

Below is the link to the electronic supplementary material.

Supplementary file1 (DOCX 36 KB)

## Data Availability

Publicly available datasets were analysed in this study. These data can be found here: https://data.europa.eu/data/datasets/the-efsa-comprehensive-european-food-consumption-database?locale=en; https://nevo-online.rivm.nl/; http://ghdx.healthdata.org/gbd-results-tool. In addition, a copy of the Dutch Food Consumption Survey (FCS) 2012–2016 was requested via https://www.wateetnederland.nl/publicaties-en-datasets/datasets, and was used to initiate the linkage between FoodEx2 and food composition data, i.e. NEVO, the Dutch Food Composition Table.

## Code availability

STATA DO-files are available on request.
