# Supplementary material for: Ultra-processed food consumption in adults across Europe
Source: Eur J Nutr. 2021 Dec 3;61(3):1521–39. doi: 10.1007/s00394-021-02733-7 (PMC8921104; doi:10.1007/s00394-021-02733-7)
Supplement: Supplementary file 1 — Supplementary file1 (DOCX 36 KB) [file 394_2021_2733_MOESM1_ESM.docx]

SUPPLEMENTARY MATERIALS

**Supplementary Table 1** Classification of the foods as coded by the Exposure Hierarchy FoodEX2 according to the NOVA classification [1,2].

| **FoodEx2 L1 Food group** | **FoodEx2 L2 Food subgroup** | **NOVA classification** |
| --- | --- | --- |
| Grains and grain-based products | Cereals and cereal primary derivatives | Minimally processed |
|  | Bread and similar products | Processed, except sandwich, sticks, pretzels and crackers and additional bread products (like croutons) (ultra-processed) |
|  | Pasta, doughs, and similar products | Minimally processed if plain, processed if filled, gnocchi, noodles, and ultra-processed if raw doughs and pre-mixes |
|  | Fine bakery wares | Ultra-processed |
|  | Breakfast cereals | Minimally processed if cereals rolled grains, porridge and muesli, and ultra-processed if cereal bars, cereal flakes and popped cereals |
| Vegetables and vegetable products | Leavy vegetables; Sprouts, shoots and similar; Flowering brassica; Flowers used as vegetables; Stems/stalks eaten as vegetables; Bulb vegetables; Legumes with pod; Fruiting vegetables; Root and tuber vegetables (excluding starchy- and sugar-); Algae and prokaryotes organisms; Fungi, mosses and lichens; Herbs and edible flowers | Minimally processed |
|  | Processed or preserved vegetables and similar | Processed if preserved concentrated, sun-dried, fermented, mashed, salted, candied or sugar-preserved, dried or canned/jarred |
| Starchy roots or tubers and products thereof, sugar plants | Starchy roots and tubers; Starchy roots and tuber products; Sugar plants | Minimally processed, except if canned (processed) |
| Legumes, nuts, oilseeds and spices | Legumes | Minimally processed |
|  | Nuts, oilseeds, and oilfruits | Minimally processed |
|  | Spices | Minimally processed |
|  | Processed legumes, nuts, oilseeds and spices | Processed if candied or sugar preserved, dried, canned or jarred, and ultra-processed if nut/seeds paste/emulsion/mass (like peanut butter, nut or seeds spreads) |
| Fruit and fruit products | Fruit used as fruit | Minimally processed |
|  | Processed fruit products | Minimally processed if dried, except fruit chips and chocolate coated dried fruit (ultra-processed), processed if fruit/vegetable spread and similar, canned or jarred, fermented, marinated or candied. |
| Meat and meat products | Animal carcase; Animal liver; Animal kidney; Animal blood; Animal other organs; Animal other slaughtering products | Minimally processed |
|  | Mammals and birds meat | Minimally processed |
|  | Animal fresh fat tissues; Preserved fat tissues | Culinary ingredient, except cured pork fat (processed) |
|  | Processed whole meat products (raw/cooked cured or seasoned meat); Animal meat dried; Marinated meat; canned-tinned meat | Processed |
|  | Sausages; Meat specialities (meat spread/pate); Edible casings; Animal mechanically separated meat | Ultra-processed |
| Fish, seafood, amphibians, reptiles and invertebrates | Fish (meat); Fish offal | Minimally processed |
|  | Crustaceans; Molluscs; Sea-squirts and other tunicates; Sea urchins and other echinoderms; Jellyfishes and similar; Amphibians, reptiles, snails, insects | Minimally processed |
|  | Fish and seafood processed | Processed if marinated/pickled, salt-preserved, canned/jarred or smoked, else ultra-processed if fish fingers, fish balls or fish paste or surimi |
| Milk and Sweetened/flavoured dairy products | Milk, whey, and cream; Fermented milk or cream | Minimally processed, except whey, sweetened or flavoured (ultra-processed) |
|  | Milk and dairy powders and concentrates | Minimally processed |
|  | Cheese | Processed, except cheese spreads (ultra-processed) |
|  | Dairy desserts and similar | Ultra-processed |
| Eggs and egg products | Unprocessed eggs | Minimally processed |
|  | Processed eggs | Minimally processed if boiled, poached, fried or powdered, and ultra-processed if manufactured egg roll |
| Sugar and similar, confectionary and water-based sweet desserts | Sugar and other sweetening ingredients (excluding intensive sweeteners); Table-top sweeteners formulations | Culinary ingredients if sugar, syrups and honey, ultra-processed if other sweetening ingredients, like polyols |
|  | Confectionary including chocolate; Water-based desserts | Ultra-processed |
| Animal and vegetable fats and oils and primary derivatives thereof | Animal and vegetable fats/oils | Culinary ingredients |
|  | Fat emulsions and blended fats | Ultra-processed, except butter (culinary ingredient) |
| Fruit and vegetable juices and nectars (including concentrates) | Fruit/vegetable juices and nectars | Minimally processed if 100% from named sources; else ultra-processed |
|  | Concentrated or dehydrated fruit/vegetables juices | Ultra-processed |
|  | Liquid or gel separated from plant RPCs | Ultra-processed |
|  | Extracts of plant origin | Culinary ingredients |
| Water and water-based beverages | Drinking water | Minimally processed |
|  | Water-based beverages; Beverages concentrates | Ultra-processed |
| Alcoholic beverages | Beer and beer-like beverages; Wine and wine-like drinks; Mixed alcoholic drinks; Unsweetened spirits and liqueurs | Not classified |
| Coffee, cocoa, tea and infusions | Coffee, cocoa, tea and herbal ingredients | Minimally processed, except cocoa ingredients (ultra-processed) |
|  | Hot drinks and similar (coffee, cocoa, tea and herbal drinks/infusion) | Minimally processed if coffee, coffee-imitate, tea, and infusions, and ultra-processed if cocoa beverages |
| Food products for young population | Infant and follow-on formulae; Ready-to-eat meal for infants and young children; Processed cereal-based food for infants and young children; Other foods for infants and children | Ultra-processed |
| Products for non-standard diets, food imitates and food supplements | Food for particular diets; Food supplements and similar preparations; Meat and dairy imitates | Ultra-processed |
| Composite dishes | Dishes, including ready to eat meals (excluding soups and salads); Soups and salads; Fried or extruded cereal or root-based products | Ultra-processed |
| Seasoning, sauces and condiments | Seasoning and extracts; Savoury extracts and sauce ingredients; Condiments (including table-top formats); Dessert sauces/toppings; Mixed and other not listed condiments | Ultra-processed, except salt (culinary ingredients) |
| Major isolated ingredients, additives, flavours, baking and processing aids | Isolated proteins and other protein products; Starches; Maltodextrines and similar; Inulin and other polyfructoses; Food colours; Food flavours; Food additives other than flavours, colours and artificial sweeteners; Miscellaneous agents for food processing | Ultra-processed |
| Other ingredients | Artificial sweeteners; Vitamins; Chemical elements; Special fatty acids; Dietary fibre; Phytochemicals; Algae based fortifying agents; Caffeine; Bee-produced fortifying agents; Co-factors to metabolism | Ultra-processed |

1. Monteiro CA, Cannon, G., Lawrence, M., Costa Louzada, M.L. and Pereira Machado, P. (2019) Ultra-processed foods, diet quality and health using the NOVA classification system. FAO, Rome

2. Monteiro CA, Cannon G, Levy RB, Moubarac J-C, Louzada MLC, Rauber F, Khandpur N, Cediel G, Neri D, Martinez-Steele E, Baraldi LG, Jaime PC (2019) Ultra-processed foods: what they are and how to identify them. Public Health Nutrition 22 (5):936-941. doi:10.1017/S1368980018003762

Supplementary Table 2 A top 5 of the ultra-processed foods consumed by European adults, as available by EFSA, ordered alphabetically and stratified by sex, expressed in percentage of daily food consumption coming from ultra-processed foods.

|  | TOP 5 |  |  |  |  |
| --- | --- | --- | --- | --- | --- |
|  | 1 | 2 | 3 | 4 | 5 |
| Eur – 22 countries | Fine bakery wares (13%) | Composite Dishes (8.5%) | Sausages (8.2%) | Sweetened/flavoured dairy products (5.9%) | Sauces (5.6%) |
| **MEN** |  |  |  |  |  |
| Eur – 22 countries | Fine bakery wares (12.2%) | Sausages (9.9%) | Composite Dishes (8.6%) | Sauces (5.7%) | Sweetened/flavoured dairy products (4.8%) |
| Austria | Fine bakery wares (16%) | Composite Dishes (8%) | Sauces (7%) | Sausages (6%) | Chocolate (2%) |
| Belgium | Fine bakery wares (8%) | Sauces (8%) | Sweetened/flavoured dairy products (5%) | Sausages (5%) | Chocolate (3%) |
| Croatia | Sausages (24%) | Fine bakery wares (4%) | Sauces (3%) | Crackers and additional bread products (3%) | Chocolate (2%) |
| Cyprus | Fine bakery wares (17%) | Crackers and additional bread products (7%) | Sauces (4%) | Breakfast cereals (3%) | Water-/dairy-based desserts (3%) |
| Czech Republic | Sausages (20%) | Fine bakery wares (18%) | Composite Dishes (10%) | Sauces (3%) | Crackers and additional bread products (2%) |
| Denmark | Margarine (8%) | Sausages (6%) | Sweetened/flavoured dairy products (4%) | Processed meat (3%) | Fine bakery wares (3%) |
| Estonia | Sausages (20%) | Fine bakery wares (7%) | Sweetened/flavoured dairy products (7%) | Sauces (6%) | Water-/dairy-based desserts (3%) |
| Finland | Sweetened/flavoured dairy products (17%) | Fine bakery wares (12%) | Sausages (11%) | Margarine (8%) | Sauces (7%) |
| France | Fine bakery wares (21%) | Sweetened/flavoured dairy products (13%) | Sauces (11%) | Sausages (6%) | Crackers and additional bread products (3%) |
| Germany | Composite Dishes (13%) | Fine bakery wares (10%) | Sausages (9%) | Sauces (6%) | Sweetened/flavoured dairy products (5%) |
| Greece | Fine bakery wares (20%) | Crackers and additional bread products (9%) | Sauces (9%) | Sausages (4%) | Water-/dairy-based desserts (3%) |
| Hungary | Sausages (21%) | Fine bakery wares (3%) | Margarine (3%) | Crackers and additional bread products (3%) | Sauces (2%) |
| Ireland | Composite Dishes (14%) | Fine bakery wares (12%) | Sauces (10%) | Breakfast cereals (6%) | Sausages (5%) |
| Italy | Fine bakery wares (23%) | Sausages (10%) | Water-/dairy-based desserts (7%) | Composite Dishes (7%) | Sweetened/flavoured dairy products (6%) |
| Latvia | Composite Dishes (23%) | Fine bakery wares (19%) | Sausages (14%) | Sauces (11%) | Sweetened/flavoured dairy products (5%) |
| The Netherlands | Composite Dishes (11%) | Fine bakery wares (7%) | Sweetened/flavoured dairy products (6%) | Sauces (6%) | Sausages (3%) |
| Portugal | Fine bakery wares (13%) | Composite Dishes (4%) | Sausages (4%) | Water-/dairy-based desserts (4%) | Sweetened/flavoured dairy products (3%) |
| Romania | Sausages (15%) | Composite Dishes (14%) | Crackers and additional bread products (4%) | Fine bakery wares (3%) | Processed meat (2%) |
| Slovenia | Fine bakery wares (21%) | Sausages (19%) | Sauces (8%) | Crackers and additional bread products (3%) | Processed meat (2%) |
| Spain | Fine bakery wares (16%) | Sweetened/flavoured dairy products (12%) | Sausages (7%) | Crackers and additional bread products (6%) | Sauces (4%) |
| Sweden | Composite Dishes (38%) | Fine bakery wares (7%) | Sauces (7%) | Sausages (5%) | Sweetened/flavoured dairy products (4%) |
| United Kingdom | Composite Dishes (29%) | Fine bakery wares (6%) | Sauces (5%) | Sweetened/flavoured dairy products (4%) | Sausages (3%) |
| **WOMEN** |  |  |  |  |  |
| Eur – 22 countries | Fine bakery wares (14.1%) | Composite Dishes (8.4%) | Sweetened/flavoured dairy products (7.1%) | Sausages (6.3%) | Sauces (5.4%) |
| Austria | Fine bakery wares (19%) | Composite Dishes (10%) | Sauces (9%) | Sausages (4%) | Chocolate (3%) |
| Belgium | Fine bakery wares (11%) | Sauces (8%) | Sweetened/flavoured dairy products (7%) | Sausages (4%) | Chocolate (3%) |
| Croatia | Sausages (15%) | Fine bakery wares (6%) | Water-/dairy-based desserts (4%) | Crackers and additional bread products (3%) | Chocolate (3%) |
| Cyprus | Fine bakery wares (21%) | Breakfast cereals (6%) | Crackers and additional bread products (5%) | Water-/dairy-based desserts (5%) | Sweetened/flavoured dairy products (4%) |
| Czech Republic | Fine bakery wares (19%) | Sausages (10%) | Composite Dishes (10%) | Sauces (3%) | Crackers and additional bread products (3%) |
| Denmark | Margarine (7%) | Sweetened/flavoured dairy products (4%) | Sausages (4%) | Candies, confectionary (3%) | Chocolate (3%) |
| Estonia | Sweetened/flavoured dairy products (15%) | Sausages (13%) | Fine bakery wares (11%) | Sauces (5%) | Chocolate (4%) |
| Finland | Sweetened/flavoured dairy products (21%) | Fine bakery wares (13%) | Margarine (8%) | Composite Dishes (7%) | Sausages (6%) |
| France | Fine bakery wares (22%) | Sweetened/flavoured dairy products (17%) | Sauces (11%) | Sausages (5%) | Crackers and additional bread products (4%) |
| Germany | Composite Dishes (16%) | Fine bakery wares (13%) | Sweetened/flavoured dairy products (8%) | Sauces (8%) | Sausages (5%) |
| Greece | Fine bakery wares (24%) | Crackers and additional bread products (14%) | Sauces (5%) | Breakfast cereals (5%) | Sweetened/flavoured dairy products (4%) |
| Hungary | Sausages (16%) | Sweetened/flavoured dairy products (5%) | Fine bakery wares (5%) | Margarine (4%) | Crackers and additional bread products (3%) |
| Ireland | Fine bakery wares (13%) | Composite Dishes (13%) | Sauces (9%) | Sweetened/flavoured dairy products (8%) | Breakfast cereals (5%) |
| Italy | Fine bakery wares (22%) | Sweetened/flavoured dairy products (13%) | Composite Dishes (7%) | Water-/dairy-based desserts (7%) | Sausages (6%) |
| Latvia | Fine bakery wares (24%) | Composite Dishes (19%) | Sauces (12%) | Sweetened/flavoured dairy products (8%) | Sausages (8%) |
| The Netherlands | Composite Dishes (10%) | Fine bakery wares (8%) | Sweetened/flavoured dairy products (6%) | Sauces (6%) | Sausages (3%) |
| Portugal | Fine bakery wares (16%) | Water-/dairy-based desserts (5%) | Composite Dishes (5%) | Sweetened/flavoured dairy products (4%) | Breakfast cereals (3%) |
| Romania | Composite Dishes (12%) | Sausages (10%) | Crackers and additional bread products (5%) | Fine bakery wares (4%) | Breakfast cereals (2%) |
| Slovenia | Fine bakery wares (28%) | Sausages (9%) | Sauces (8%) | Sweetened/flavoured dairy products (6%) | Crackers and additional bread products (6%) |
| Spain | Fine bakery wares (14%) | Sweetened/flavoured dairy products (12%) | Crackers and additional bread products (6%) | Sausages (5%) | Sauces (3%) |
| Sweden | Composite Dishes (38%) | Fine bakery wares (7%) | Sauces (7%) | Sweetened/flavoured dairy products (5%) | Sausages (3%) |
| United Kingdom | Composite Dishes (26%) | Fine bakery wares (7%) | Sauces (7%) | Sweetened/flavoured dairy products (5%) | Breakfast cereals (3%) |

*Abbreviation:* Eur, Europe

**Supplementary Table 3** A top 3 of the ultra-processed drinks consumed by European adults, ordered alphabetically and stratified by sex, expressed in percentage of daily food consumption coming from ultra-processed drinks

|  | TOP 3 |  |  |
| --- | --- | --- | --- |
|  | **1** | **2** | **3** |
| Eur – 22 countries | Soft drinks (29%) | Fruit/vegetables juices not 100% from named source (4.9%) | Diet soft drinks (2.9%) |
| **MEN** |  |  |  |
| Eur – 22 countries | Soft drinks (32.6%) | Fruit/vegetables juices not 100% from named source (4.3%) | Diet soft drinks (2.6%) |
| Austria | Soft drinks (38%) | Fruit/vegetables juices not 100% from named source (5%) | Diet soft drinks (5%) |
| Belgium | Soft drinks (42%) | Diet soft drinks (15%) | Sweetened/flavoured milk (1%) |
| Croatia | Soft drinks (42%) | Fruit/vegetables juices not 100% from named source (12%) | Sweetened/flavoured milk (0%) |
| Cyprus | Soft drinks (44%) | Fruit/vegetables juices not 100% from named source (5%) | Milk imitates (1%) |
| Czech Republic | Soft drinks (33%) | Fruit/vegetables juices not 100% from named source (6%) | Soups (0.5%) |
| Denmark | Soft drinks (52%) | Sweetened/flavoured milk (6%) | Fruit/vegetables juices not 100% from named source (2%) |
| Estonia | Soft drinks (28%) | Fruit/vegetables juices not 100% from named source (7%) | Sweetened/flavoured milk (5%) |
| Finland | Soft drinks (14%) | Diet soft drinks (7%) | Cocoa beverages (3%) |
| France | Soft drinks (30%) | Fruit/vegetables juices not 100% from named source (2%) | Cocoa beverages (2%) |
| Germany | Soft drinks (29%) | Soups (9%) | Fruit/vegetables juices not 100% from named source (6%) |
| Greece | Soft drinks (34%) | Fruit/vegetables juices not 100% from named source (10%) | Diet soft drinks (1%) |
| Hungary | Soft drinks (45%) | Sweetened/flavoured milk (13%) | Diet soft drinks (2%) |
| Ireland | Soft drinks (30%) | Diet soft drinks (2%) | Fruit/vegetables juices not 100% from named source (2%) |
| Italy | Soft drinks (24%) | Fruit/vegetables juices not 100% from named source (11%) | Soups (0.4%) |
| Latvia | Soft drinks (15%) | Fruit/vegetables juices not 100% from named source (4%) | Sweetened/flavoured milk (0.1%) |
| The Netherlands | Soft drinks (31%) | Diet soft drinks (13%) | Sweetened/flavoured milk (6%) |
| Portugal | Soft drinks (39%) | Sweetened/flavoured milk (10%) | Fruit/vegetables juices not 100% from named source (9%) |
| Romania | Soft drinks (35%) | Soups (17%) | Fruit/vegetables juices not 100% from named source (1%) |
| Slovenia | Soft drinks (27%) | Fruit/vegetables juices not 100% from named source (3%) | Sweetened/flavoured milk (2%) |
| Spain | Soft drinks (34%) | Milk imitates (6%) | Fruit/vegetables juices not 100% from named source (3%) |
| Sweden | Soft drinks (19%) | Soups (5%) | Diet soft drinks (3%) |
| United Kingdom | Soft drinks (31%) | Diet soft drinks (5%) | Soups (5%) |
| **WOMEN** |  |  |  |
| Eur – 22 countries | Soft drinks (25.7%) | Fruit/vegetables juices not 100% from named source (5.6%) | Sweetened/flavoured milk (3.4%) |
| Austria | Soft drinks (29%) | Fruit/vegetables juices not 100% from named source (7%) | Diet soft drinks (5%) |
| Belgium | Soft drinks (29%) | Diet soft drinks (21%) | Milk imitates (3%) |
| Croatia | Soft drinks (39%) | Fruit/vegetables juices not 100% from named source (16%) | Sweetened/flavoured milk (1%) |
| Cyprus | Soft drinks (30%) | Fruit/vegetables juices not 100% from named source (6%) | Milk imitates (4%) |
| Czech Republic | Soft drinks (30%) | Fruit/vegetables juices not 100% from named source (14%) | Sweetened/flavoured milk (0.3%) |
| Denmark | Soft drinks (53%) | Sweetened/flavoured milk (8%) | Fruit/vegetables juices not 100% from named source (2%) |
| Estonia | Soft drinks (18%) | Fruit/vegetables juices not 100% from named source (8%) | Sweetened/flavoured milk (6%) |
| Finland | Soft drinks (8%) | Diet soft drinks (6%) | Cocoa beverages (2%) |
| France | Soft drinks (24%) | Fruit/vegetables juices not 100% from named source (4%) | Cocoa beverages (2%) |
| Germany | Soft drinks (18%) | Soups (11%) | Fruit/vegetables juices not 100% from named source (8%) |
| Greece | Soft drinks (26%) | Fruit/vegetables juices not 100% from named source (10%) | Diet soft drinks (1%) |
| Hungary | Soft drinks (35%) | Sweetened/flavoured milk (17%) | Diet soft drinks (2%) |
| Ireland | Soft drinks (25%) | Diet soft drinks (7%) | Fruit/vegetables juices not 100% from named source (2%) |
| Italy | Soft drinks (18%) | Fruit/vegetables juices not 100% from named source (12%) | Soups (1%) |
| Latvia | Soft drinks (9%) | Fruit/vegetables juices not 100% from named source (6%) | Sweetened/flavoured milk (1%) |
| The Netherlands | Soft drinks (24%) | Diet soft drinks (17%) | Sweetened/flavoured milk (8%) |
| Portugal | Soft drinks (25%) | Sweetened/flavoured milk (16%) | Fruit/vegetables juices not 100% from named source (8%) |
| Romania | Soft drinks (37%) | Soups (18%) | Fruit/vegetables juices not 100% from named source (1%) |
| Slovenia | Soft drinks (16%) | Fruit/vegetables juices not 100% from named source (5%) | Sweetened/flavoured milk (4%) |
| Spain | Soft drinks (34%) | Milk imitates (9%) | Sweetened/flavoured milk (2%) |
| Sweden | Soft drinks (14%) | Soups (6%) | Diet soft drinks (4%) |
| United Kingdom | Soft drinks (27%) | Diet soft drinks (7%) | Soups (6%) |

*Abbreviation:* Eur, Europe
